# The Role of Family Support and Dyadic Adjustment on the Psychological Well-being of Transgender Individuals: An Exploratory Study

**DOI:** 10.1007/s13178-023-00817-z

**Published:** 2023-05-08

**Authors:** Jessica Lampis, Silvia De Simone, Diego Lasio, Francesco Serri

**Affiliations:** Department of Education, Psychology, Philosophy, University of Cagliari, Via Is Mirrionis, 1, 09123 Cagliari, Italy

**Keywords:** Transgender individuals, Psychological well-being, Social support, Family support, Dyadic adjustment

## Abstract

**Introduction:**

This study aimed to measure dyadic adjustment, social support, and psychological well-being.

**Methods:**

A research protocol composed of the Dyadic Adjustment Scale, the Outcome Questionnaire 45.2, and the Multidimensional Scale of Perceived Social Support was administered to a sample of 109 Italian transgender individuals.

**Results:**

Higher levels of global psychological distress, symptom severity, and interpersonal relationship distress were associated with lower levels of family support and dyadic adjustment. In addition, transgender women and younger transgender individuals reported higher levels of interpersonal relationship distress.

**Conclusions:**

The results indicate that the support and acceptance of one’s partner and family of origin play a crucial role in promoting well-being. It represents an important protective factor with respect to negative psychological health outcomes.

**Policy Implications:**

The findings emphasize the need to develop specific clinical and social practices for transgender individuals and their families. Building family and partner-centered policies and programs is particularly important to enable transgender individuals to avoid paying the emotional and psychological costs associated with rejection and non-acceptance.

## Introduction

Transgender individuals form a gender minority that frequently faces stigma and discrimination due to a society that remains fundamentally heteronormative and based on gender binarism that legitimizes only two sex categories and characterizes variants as non-normal (Butler, 2004; Dierckx et al., 2017; Walch et al., 2012).

The postulate that “men are from Mars and women are from Venus” still pervades popular culture (Iantaffi & Bockting., 2011) and leads to confusion or the interchangeable use of sex and gender. Furthermore, encompasses sexuality in the performance of normative masculinity and femininity (Figueira, 2016) and culturally constructs a view of transgenderism as a problematic deviation from the norm (Norwood, 2012).

In recent years, the conditions and needs of transgender individuals have become increasingly visible (European Union Agency for Fundamental Rights, 2014); however, heteronormative expectations remain a challenge for them and their families (Israel, 2005).

In Italy, for example, in the context of a progressive access of LGBTQ + people to formal institutions, sexual policies are marked by mixed trends: despite the recently passed law that recognizes same-sex couples as having most of the rights of married heterosexual couples (L.76/2016), heterocisnormativity remains hegemonic (Lasio et al., 2019).

Emblematic, in this regard, was, in February 2022, the rejection in the Senate of the Zan DDL “Measures for the prevention and contrast of discrimination and violence on grounds of sex, gender, sexual orientation, gender identity and disability.” The proposed new law would seek to extend the protections of the 1993 Mancino Law by amending the penalties under Article 604-bis of the Criminal Code for those who “incite to commit or commit acts of discrimination on racial, ethnic, national or religious grounds,” adding “or based on sex, gender, sexual orientation, gender identity or disability.” Among the goals of the law is also the establishment of the “National Day Against Homophobia, Lesbophobia, Biphobia and Transphobia,” to be celebrated on May 17.

The heated political and institutional debate that led to the rejection of the Zan DDL is an expression of a strong social and cultural movement in our country that continues to deny, reject, disqualify, and stigmatize individual and relational experiences that do not fit into the social order.

In our country, moreover, trans experience (in the paper “transgender” and “trans” as an adjective will be used interchangeably) still seems to be conceived as a pathological expression of the self. While the last decade of the twentieth century has seen the emergence of a new model that considers transgenderism as a natural form of human variability (Denny et al., 2004), in Italy, the medical model that considers it a form of mental illness still prevails.

The Law 164/82, in fact, established the forced medicalization of trans people through the institutionalization of the medical-legal consultation required to start the gender transition process (Voli, 2018), thus denying trans people the possibility of self-determination and subordinating the transition pathway to the opinion of professional figures (Giardina & Zabonati, 2020).

More recently, a sentence of the Supreme Court of Cassation (ruling act no. 15138/15) has established that surgical interventions should not be considered strictly necessary for having one’s gender identity legally recognized, but the official legislation dating back to 1982 has never been formally changed (Scandurra et al., 2023).

In Italy, the medical paradigm is deeply entrenched in the sexual and gender binary model, forcing trans people to undergo physical correction of characteristics that are considered abnormal because they subvert the correspondence between sex and gender (Giardina & Zabonati, 2020).

Although recent Italian studies have revealed that the experiences of transgender people do not align with the medical model and that psychological distress is determined by demographic and contextual factors (Anzani et al., 2020; Golbach et al., 2022), the gender affirmation pathways are still seen as intrinsically characterized by psychic suffering, while the discomfort due to the absence of social recognition or the internalization of transphobic and cisnormative social norms is neglected (Pieraccini, 2013).

This extremely narrow focus on mental health outcomes may result in the over-pathologization of a vulnerable population that may experience normative responses to pervasive discrimination, violence, and exclusion. Furthermore, the emphasis placed exclusively on individual problems denotes the risk of the continued neglect of relational, social, and political contexts from which suffering emerges, acquires significance, and can be treated (Lasio et al., 2020a, b). Therefore, it is important to focus on other very important aspects of the lives of transgender individuals, especially their daily lives and, in particular, their families and relational world.

Starting from these assumptions, the study investigates the presence of a relationship of psychological distress with experiences of discrimination, marginalization, and victimization (Hendricks & Testa, 2012; Lefevor et al., 2019; Puckett et al., 2020) and stresses the importance of identifying the needs of transgender individuals in terms of mental health interventions (Riggs et al., 2015a, b; Riggs et al., 2015a, b).

Today, the need to include an evaluation of the contextual factors that produced vulnerability for mental health symptoms is well established in the assessment of these conditions. A common knowledge is that transgender individuals at all stages of transition (i.e., medical and social) report difficulties in interpersonal relationships (Nobili et al., 2018; Stewart et al., 2018) and challenges within family dynamics (Dierckx et al., 2017).

A recent review (Valentine & Shipherd, 2018) has revealed that depressive symptoms, anxiety, substance use disorders, and suicidality are consistently elevated among transgender and gender-non-conforming people. Furthermore, the analysis of risk and resilience factors revealed that discrimination and violence are major distal minority stressors; expectations of rejection, identity concealment, and internalized stigma (transphobia) are major proximal minority stressors; finally, coping skills, social support from peers and family, and gender minority communities are the main interactive proximal resilience factors.

Recent studies conducted in Italy partially confirm this evidence. Recent research (Anzani et al., 2020) aimed at exploring personality disorders and personality profiles in a sample of transgender individuals requesting gender-affirming treatments showed that, using the categorical approach to PDs diagnosis through structured clinical interviews, nearly half of transgender clients had at least one PD diagnosis. Another study (Mirabella et al., 2022) analyzing the psychological well-being of trans people in Italy during the COVID-19 pandemic revealed that respondents reported high levels of depression, hostility, anxiety, and discouragement about the future, and they reported negative changes in psychological well-being between the period before the outbreak and the period throughout the emergency.

These results offer insights into the effects of growing up and living in discriminating and stigmatizing environments. Transgender and gender-non-conforming people are more likely than cisgender people to experience physical, psychological, and sexual violence, discrimination, harassment, and rejection by friends.

Other Italian studies (Scandurra et al., 2017, 2018) also confirm that transgender individuals are significantly burdened by existing mental health problems; however, anti-transgender discrimination and internalized transphobia are positively associated with negative mental health outcomes, and resilience is negatively associated with mental health problems (Scandurra et al., 2018). Furthermore, younger transgender individuals are more likely to experience mental health problems than older cohorts (Scandurra et al., 2023), perhaps due to less extensive and weaker social support networks.

The reason for exploring transgender families in this manner is twofold: first, research has demonstrated that the family context can exert a positive influence on health and well-being; second, the family context can be an important factor in the process of finding appropriate support for transgender individuals (Testa et al., 2015).

Moreover, other issues, such as family support, access to partnering, and parenting rights, which include marriage, civil partnership, and adoption, acquired center stage in struggles around equality policies and sexual freedom. Concepts, such as *intimate citizenship* (Plummer, 2003) and *the sexual citizen* (Bell & Binnie, 2000), became increasingly popular given that they are grounded in the daily experiences of people living and loving beyond the heteronorm (Lasio et al., 2020a, b; Roseneil, 2005).

Analysis of the literature on transgender individuals revealed that articles that addressed the experiences of family members, friends, and romantic partners are few (Erich et al., 2008; Ryan et al., 2010). To address this research gap, the current study moves from the main objectives to provide cross-sectional evidence of the association between resilience factors, such as social support from family and significant other, dyadic adjustment, and psychological well-being, in a sample of transgender individuals.

## Partner Relationships and Well-being

The couple relationship seems to be a powerful protective factor for the psychological well-being of LGBTQ individuals. Recent studies (Riggle et al., 2010; Hatzenbuehler et al., 2011; Cherlin, 2013) have revealed that gay and lesbian individuals in legally and socially recognized relationships experience fewer depressive symptoms and lower stress levels than those who are not recognized. Confirming these observations, the self-perceived health of gay and lesbian people is also deemed significantly lower than that perceived by heterosexual people and varies significantly in relation to the legal recognition of their affective and relational realities (Liu et al., 2013; Kail et al., 2105).

In recent years, a paucity of research on the couple relationships of transgender individuals has been observed in contrast to couples composed of gay and lesbian individuals (Platt & Bolland, 2017). Studies on the couple relationships of transgender individuals and their partners have modestly increased; there is still very little research available (Bischof et al., 2016). Moreover, research on transgender relationships mainly focuses on the romantic partners of transgender individuals and fails to center on transgender individuals themselves (Joslin-Roher & Wheeler, 2009; Norwood, 2013). Although the focus on romantic partners provides meaningful insight into one side of the relationship, it keeps the identity of the (cisgender) partner in the foreground, thus continuing the stigma that transgender individuals are a burden not only to society but also to their romantic partners. The experiences of partners are a valuable contribution to the literature, but further research is needed to directly examine the experiences of transgender individuals in relationships (Pulice-Farrow et al., 2019).

The following is a brief discussion of what the more recent studies have found about couples in which at least one partner is transgender.

Although the relationship of couples in which at least one partner is a transgender person is a unique experience influenced by a multitude of factors, research identifies some common processes and daily challenges within these relationships (Platt et al., 2018; Marshall et al., 2020).

Research has identified a number of common themes: difficulties in finding a partner (Belawski & Sojka, 2014); the risk associated with the phase of unveiling one’s identity to the partner (Platt & Bolland, 2017); the changes the couple faces in daily life and their effect on dyadic adaptation (Meier et al., 2013; Platt & Bolland, 2017); and the role of minority stress on couple functioning and partners’ psychological well-being (Gamarel et al., 2014, 2019).

The body of research on transgender individuals’ romantic relationships has focused on the difficulty of finding a partner due to the risk of interpersonal rejection that may follow the revelation of one’s trans identity (Belawski & Sojka, 2014; Mizock & Mueser, 2014). Although transgender individuals fall in love as much as cisgender individuals, they have significantly fewer sexual and romantic experiences than their cisgender peers (Bungener et al., 2017).

The framework offered by the research reveals that the experiences of transgender woman are much less positive and focuses mainly on risks perceived when they meet possible cisgender partners (Lewins, 2002). Transgender women seemingly undergo more difficulty than transgender men do to experience romantic relationships (Riggs et al., 2015a, b), and they are much more distressed during the process of revealing their identity due to the fear of rejection (Iantaffi & Bockting, 2011). This difference may be due to the higher level of stigmatization reported by transgender women (Baams et al., 2015; Roberts et al., 2014), which may prevent them from entering into romantic relationships due to fear of the disclosure of their identity and the reactions of potential partners (Marshall et al., 2020).

Another particularly critical moment is the revelation of trans identity to the partner, which may be a long-standing one. This phase is typically a life-changing and deeply personal experience for both sides (Platt & Bolland, 2017). For transgender individuals, the experience of disclosure with a partner can be related to specific themes, such as the obligation to disclose, the unpredictability of the disclosure process and of others’ responses, and the feeling of liberation after the disclosure (Bethea & McCollum, 2013). Partners may perceive that they are being asked to change their sexual orientation based on the gender identity of their trans partners (Theron & Collier, 2013; Whitley, 2013) or may be concerned about the stigma that may accompany being in an intimate relationship with a trans individual. The risks associated with this type of disclosure are relatively significant due to the imminent possibility of losing one’s family, marriage, and children (Dierckx et al., 2019; Flynn, 2006; Samons, 2009).

The acceptance of the partner can also be influenced by the way the trans partner comes out. When disclosure involves a gradual process, the cis partner often expresses more understanding. If the disclosure occurs abruptly and suddenly, or if the trans partner does not reveal his or her gender identity, but the cis partner discovers it, this is likely to result in greater discomfort and emotional distress (Bischof et al., 2011; Dierckx et al., 2019).

Studies have also focused on the profound changes that often occur in the dyadic adjustment, satisfaction, and sexuality of partners during and after transition Marshall et. al. 2020; Platt & Bolland, 2017). Transition often leads to feelings of loneliness and isolation that can negatively affect relationship satisfaction; therefore, maintaining clear and open communication and sexual intimacy can ensure a healthy relationship that in turn improves relationship quality and satisfaction (Platt & Bolland, 2018).

In a study exclusively involving trans men, approximately half of the relationships were unsuccessful during or after transition (Meier et al., 2013). However, although reactions to the disclosure of a person’s trans identity may include confusion or feelings of betrayal, many partners may feel that the revelation can actually strengthen their relationship (Joslin-Roher & Wheeler, 2009). Trans participants who remained in their previous relationships reported less depression and anxiety and indicated that they obtained increased social support (Meier et al., 2013). Furthermore, personal resilience and the number of relationship years prior to transition predict relational commitment and relationship satisfaction for cisgender individuals paired with a self-identified transgender individual (Platt, 2020).

Finally, studies conducted in this field have revealed that discrimination, marginalization, stigma, economic difficulties, and poor social support can cause distress, generate interpersonal conflicts, and be associated with a perception of low relational quality (Bodenmann et al., 2007; Constantinides, 2012; Gamarel et al., 2014; Karney et al., 2005).

The experience of minority stressors within couple relationships negatively affects the quality of the relationship and the mental health of both the transgender individual and the partner (Iantaffi & Bockting, 2011; Newcomb, 2020; Platt & Bololand, 2017). Relational commitment and strong personal and social support networks, on the other hand, can reduce associations between interpersonal stigma and psychological distress and help partners improve their relationship satisfaction (Fuller & Riggs, 2021; Gamarel et al., 2019).

These results suggest that remaining in an intimate relationship with a partner may help a trans person feel worthy and loved after taking the risk of revealing their identity. Being in a relationship during transition and the support of a partner can contribute to improving the general well-being of transgender people during social transition (Marshall et al., 2020). As such, reflecting on these issues is very important for the transgender community given the importance and significance of couple relationships to mental and physical health in general (Gamarel et al., 2014; Lampis et al., 2021; Sanger, 2010). This latest evidence also revealed that the stressors faced by couples in which one or both partners are transgender are, therefore, innumerable and daily, and their management becomes increasingly complex if the couple is isolated or lacks quality family and social support.

## Family and Social Support and Well-being

Trans identities are significant not only for transgender individuals but also for their families. The meanings constructed by family members for stressors, particularly those involving social stigma, are important for their emotional well-being and their acceptance of the stigmatized member (Drydakis, 2022).

The Gender Minority Stress and Resilience Model is developed specifically for transgender individuals and illustrates that minority stressors, such as gender-related discrimination, victimization, and rejection, negatively impact health and well-being. Conversely, resilience factors, such as community connection, social support, and pride, can serve as a buffer against other stressors (Hendricks & Testa, 2012; Testa et al., 2015).

Perceived support specifically related to one’s sexual identity may be particularly beneficial for LGBTQ individuals (Doty et al., 2010). Furthermore, peer support and support from family and friends are deemed important sources of help and a strong protective factor against social exclusion and prejudice especially for transgender individuals or partners (Bischof et al., 2011; Joslin-Roher & Wheeler, 2009). The support of parents largely influences the psychological well-being of transgender individuals (Ishii, 2018). However, despite the exponential increase in studies that examine transgender issues, research conducted on this topic is few (Kavanaugh et al., 2019) and several of them specifically highlight transgender youth. Quantitative findings indicate that family support is associated with living in one’s affirmed gender identity, positive mental health outcomes, and higher quality of life among transgender youth. Other studies propose that transgender youth appear to experience moderate and higher levels of support from family and from peers and significant others, respectively (McConnell et al., 2015; Weinhardt et al., 2019). This finding is consistent with the notion that people choose their friends and significant others based on acceptance, shared values, and interests, but a larger variation is observed in terms of family support for gender non-conformity.

In a sample of young people receiving care in a gender clinic, Simons et al. (2013a) found that higher levels of perceived parental support were associated with fewer symptoms of depression and better overall quality of life (Simons et al., 2013a, b). Resilience, parental closeness, and parental acceptance were also protective factors against negative mental health outcomes in a sample of transfeminine youth of color (Le et al., 2016). These studies suggest that parental support is one of the many important factors, such as resilience and social context, that impact the health outcomes of transgender youth (Alanko & Lund, 2020). Furthermore, McConnell et al. (2015) compared the levels of support of a friend or a significant other with family support among transgender youth and found that those who perceive higher levels of support from friends and significant others but lower levels or absent family support remained at risk for worse mental health outcomes than those with higher levels of family support. This finding indicates that family support exerts a greater impact on the well-being of transgender youth than support from friends (Weinhardt et al., 2019).

In general samples of transgender adults, social support from family and friends is associated with less psychological distress (Budge et al., 2013) and higher levels of life satisfaction (Erich et al., 2008). For example, Seibel et al. (2018) reported that the lack of parental support increased the chance of living without permanent housing by nearly four times and decreased the self-esteem of transgender individuals. Moreover, Bradford et al. (2013) conducted a survey on transgender residents in Virginia and found that transgender individuals with higher levels of family support and those living in rural instead of suburban communities were less likely to report experiences of discrimination in housing, health care, and employment. Therefore, family and community support may be particularly important for transgender individuals in rural areas where cultural values and transgender-specific community organizations, social networks, health care, and other resources are scarce, difficult to access, or non-existent (Aaron & Rostosky, 2019).

Some studies conducted in Italy on variables that influence the psychological well-being of transgender individuals mainly emphasized the constructs of minority stress and internal transphobia (Scandurra et al., 2017, 2018, 2019). Such studies revealed that exposure to daily discrimination and internalized transphobia are associated with increased mental health problems, whereas resilience and perceived social support from family significantly reduced the strength of the association between daily discrimination and mental health (Scandurra et al., 2017). Moreover, internalized transphobia emerged as a mediator between anti-transgender discrimination and mental health, whereas resilience was the individual-level coping mechanism that buffers this relationship (Scandurra et al., 2018). Lastly, family rejection, visibility of the body, the negative effects of family violence on health, and the integration of transgender and gender-non-conforming identity were the main themes observed in the narratives on subjective experiences related to minority stress (Scandurra et al., 2019).

Other studies in Italy that refer to attachment theory were conducted (Amodeo et al., 2015; Cussino et al., 2017, 2019). The results revealed that 92.3% of patients attending the Centro Interdipartimentale Disturbi Identità di Genere and administering Adult Attachment Interview exhibited an insecure attachment state of mind (Cussino et al., 2019). Moreover, nearly half of the sample reported one or more traumatic life events with no differences found between the AMAB and AFAB groups (Cussino, et al., 2017, 2019).

In addition, studies revealed that transgender participants with secure attachment styles reported higher levels of positive transgender identity than those with insecure attachment styles. Conversely, secure attachment styles significantly influenced positive transgender identity, whereas insecure attachment styles influence internalized transphobia (Amodeo et al., 2015).

## The Present Study

A paucity exists in studies conducted in Italy on the relationship between social support and mental health. To the best of our knowledge, studies on samples of transgender individuals in Italy that focused on couple relationships are lacking. To address this research gap, the main objective of the current study is to examine the association of the well-being of transgender individuals to the quality of family/social support and couple relationships in a sample of Italian transgender individuals (Erich et al., 2008).

Toward this end, the study began with an ecological and systemic perspective that conceives the *person in the environment* and emphasizes the centrality of family relationships in understanding individual functioning. The study proposes that mutually beneficial interactions with one’s family/couple system partially predict higher levels of well-being among transgender individuals. The study employs the resilience model (Testa et al., 2015) that stresses the importance of the mediating roles of partner, family, and social support on levels of psychological distress among transgender individuals.

The study hypothesized that:

- Participants engaged in a couple relationship have lower levels of psychological distress than singles.
- Higher levels of dyadic adjustment predict lower levels of psychological distress, whereas higher levels of social support predict lower levels of psychological distress.

## Method

### Participants

The sample included 109 individuals out of which 37 (34.3%) defined themselves as transgender women and 64 (59.2%) as transgender men, whereas 7 (6.5%) provided no response and were excluded from the analysis. The age ranged from 18 to 59 years (*M* = 30.75 years). In terms of sexual orientation, 8 (6.7%) identified themselves as gay, 3 (2.5%) as lesbian, 18 (15.1%) as bisexual, 36 (30.3%) as heterosexual, 17 (14.3%) as pansexual, and 20 (16.8%) as other categories (e.g., demisexual, asexual, bi-romantic, panromantic, and questioning). In terms of relationships, 2.5% of the respondents were married, 18.5% were cohabiting but unmarried, 19.3% were in a stable relationship but unmarried or cohabiting, and 45.4% were single. The lengths of the relationship ranged from 1 to 49 years (*M* = 6.75 years). Lastly, 72.3% and 10.9% were without children and at least one child, respectively.

With regard to the level of education, 3.4% held PhD degrees, 19.3% held master’s degrees, 37.8% graduated from high school, 6.7% held a professional qualification, 16.8% held middle school certificates, 1.7% held primary school certificates, and 14.3% provided no response. Lastly, according to profession, 21.8% and 48.7% are students and employees, respectively, whereas the remainder is unemployed.

### Procedure

The study was conducted online via a survey distributed to consenting adults who identify as transgender. Data were collected through secure Google survey models in addition to online advertisements sent by the authors via mail and messages to associations in Italy that are interested in or work with LGBTQ + issues. The study requested these organizations to send the “link” for the questionnaire to their members and supporters. In this manner, the study engaged in “snowball” sampling to recruit participants. The criteria for inclusion required potential participants to identify as transgender and to be more than 18 years of age. The respondents provided consent to participate on the first page of the survey instrument. A basic demographic questionnaire was completed on the next page of the survey. Information collected included age, level of education, profession, gender identity, sexual orientation, relationship status, length of relationship, and presence and number of children.

The study observed all appropriate ethical guidelines and received approval to conduct the study from the institutional ethics board of the Department of Psychology at the first author’s institution. Participation was voluntary, and the information provided was anonymous and confidential. The participants provided digitally signed informed consent prior to the study.

### Instruments

#### Individual and Demographic Characteristics

Participants were asked specific questions about their demographic and personal characteristics, including age, gender identity, sexual orientation, education, professional status, nationality, and relationship status.

The Dyadic Adjustment Scale (DAS; Gentili et al., 2002; Spanier, 1976) is a 32-item self-report scale that measures each partner’s adaptation within the relationship and their perception of the quality of the relationship using four subscales, namely, dyadic consensus (CON; e.g., *Please indicate below the approximate extent of agreement or disagreement between you and your partner for philosophy of life*), dyadic satisfaction (SAT; e.g., *How often do you discuss or have you considered divorce, separation, or the termination of your relationship?*), dyadic cohesion (COH; e.g., *How often do you and your mate calmly discuss something?*), and affective expression (AE; e.g., *Please indicate below the approximate extent of agreement or disagreement between you and your partner for sex relations*). The following internal consistencies have been reported: total DAS score: *α* = 0.96; CON: *α* = 0.90; SAT: *α* = 0.94; COH: *α* = 0.86; AE: *α* = 0.73 (Spanier, 1976). Although the DAS is a questionnaire created to detect dyadic adjustment in couples composed by heterosexual and cisgender partners and has been validated on samples of heterosexual and cisgender individuals, several studies have demonstrated its validity in samples of transgender individuals as well (e.g., Gamarel et al., 2014; Martins et al., 2016; Tornello, 2020).

We used only total DAS scores (0–151) in our analyses. In the present study, the internal consistency reliability calculated with Cronbach’s alpha was 0.91.

The Multidimensional Scale of Perceived Social Support (MSPSS; Prezza & Principato, 2002; Zimet et al., 1988) is a 12-item self-report measure of perceived social support and is designed to assess the adequacy of support from family (e.g., *My family is willing to help me make decisions*), friends (e.g., *My friends really try to help me*), and a significant other (e.g., *There is a special person who is around when I am in need*). Items were rated using a 7-point scale, where higher scores are associated with higher levels of perceived social support. The MSPSS was found to possess good reliability (*α* = 0.81–0.98; test–retest = 0.72–0.85) and validity based on several studies (Zimet et al., 1988; Prezza & Principato, 2002).

The MSPSS has been used in several studies involving samples of transgender people (e.g., Davey et al., 2016; Tantirattanakulchai et al., 2022; Thorne et al., 2018; Trujillo et al., 2017).

In the present study, the internal consistency reliability calculated with Cronbach’s alpha was 0.94 for support for family, 0.94 for support from friends, and 0.90 for support from significant others.

The Outcome Questionnaire 45.2 (OQ-45.2; Lo Coco et al., 2006; Lambert et al., 1996) contains 45 items on which respondents specify their level of distress with a specific symptom with reference to the past week. The scores are calculated according to three subscales, namely, Symptom Distress (SD; 22 items; e.g., “*I feel no interest in things*”), Interpersonal Relations (IR; 11 items; e.g., “*I feel stressed at work/school*”), and Social Role (SR; 9 items; e.g., “*I work/study too much*”). The items were rated using a 5-point scale ranging from 0 = never to 4 = ever. Higher scores indicate higher levels of psychological distress. In the Italian version of OQ-45.2, the validation of internal consistency was reported at *α* = 0.90 for the total OQ-45.2 score, and *α* = 0.89 for SD, *α* = 0.70 for IR, and *α* = 0.61 for SR.

The OQ.45 was used by its creator Lambert in a study aimed at analyzing the psychotherapy outcomes of sexual minority clients (Mondragon, 2015) and in a recent study on longitudinal effects of psychotherapy with transgender and non-binary clients (Budge et al., 2021).

In the present study, the internal consistency reliability calculated with Cronbach’s alpha was 0.95 for the total OQ-45.2, 0.94 for SD, 0.80 for IR, and 0.77 for SR.

### Data Analyses

Statistical analyses were performed using SPSS 20. The study used descriptive statistics to analyze the socio-demographic characteristics of the sample and levels of psychological distress, social support, and dyadic adjustment. Moreover, Pearson’s product–moment correlation coefficients were used to analyze the correlations between the analyzed dimensions. A one-way univariate ANOVA was used to explore the differences between transgender single and transgender involved in a couple relationship with respect to levels of psychological distress.

Finally, the study conducted multiple linear regression analysis to investigate the predictability of psychological distress as a function of the different dimensions of social support and dyadic adjustment. The presence of multicollinearity was preliminarily investigated by inspecting the variance inflation factor (VIF) associated with each independent variable. In the present study, other assumptions of regression analysis (i.e., linear relationship, multivariate normality, no auto-correlation, and homoscedasticity) were met.

#### Psychological Distress, Social Support, and Dyadic Adjustment: Descriptive and Correlation Statistics

In the first step, a one-way univariate ANOVA was performed to explore significant differences between single and engaged in couple relationship groups with respect to levels of psychological distress. The study found that singles compared with those engaged in a couple relationship showed significantly higher levels of global psychological distress (*F*_1, 4740_ = 6.63, *p* > 0.5) and of interpersonal relation distress (*F*_*1*, 824_ = 18.22,* P* < .01).

Next, the study conducted correlation analyses to evaluate the associations between the study variables (Table 1). Correlation analyses revealed that higher levels of OQ-45.2 dimensions are significantly associated with lower levels of support from family, friends, and a significant other and with lower levels of dyadic adjustment.

**Table 1:** Psychological distress, social support, and dyadic adjustment: descriptive and correlation statistics (*n* = 102)

	Mean	S. D	Symptom distress	Intepersonal relationship	Social roles	OQ—total	Family support	Friend support	Significant other support	Dyadic adjustment
Symptoms distress	37.84	17.69	1	.707^**^	.724^**^	.967^**^	–.234^*^	–.238^*^	−.205^*^	−.407^**^
Intepersonal relationship	15.01	7.40		1	.588^**^	.824^**^	–.458^**^	−.331^**^	−.455^**^	−.642^**^
SR	14.15	6.68			1	.840^**^	–.359^**^	−.116	−.180	−.232
OQ—total	66.49	27.71				1	–.396^**^	−.189	−.265^*^	−.450^**^
Family support	15.51	5.98					1	.103	.424^**^	.347^**^
Friends support	19.31	4.16						1	.418^**^	.285^*^
Significant other support	20.55	4.00							1	.434^**^
Dyadic adjustment	113.21	18.42								1

**p < *.001*; *p < *.05*

Finally, a one-sample *t*-test was performed to compare OQ-45.2 scores with the cutoff scores reported in the Italian adaptation of the OQ-45.2 (Chiappelli et al., 2008). The study observed that the transgender persons in the sample displayed lower scores with respect to the normative clinical sample but higher scores with respect to the normative non-clinical sample (Table 2).

**Table 2:** One-sample *t*-test. Descriptors of psychological distress and comparison with normative data (Chiappelli et al., 2008)

	The current study sample (*n* = 102)		Normative non-clinical sample (*n* = 514)			Normative clinical sample (*n* = 298)		
	Mean	S. D	Mean	S. D	*t*-test	Mean	S. D	*t*-test
Symptom distress	37.84	17.69	30.77	12.51	3.89**	47.35	14.82	−5.23**
Interpersonal relationship	15.01	7.40	12.84	6.11	2.65**	17.51	6.76	−3.05*
Social roles	14.15	6.68	11.08	3.92	4.43**	15.10	5.47	−1.37
OQ—total	66.49	27.71	54.69	20.06	3.71**	79.97	23.07	−4.24**

**p < *.001*; *p < *.05*

#### How Dyadic Adjustment and Support From Family, Friends, and a Significant Other Predict Levels of Psychological Distress

The study conducted four rounds of linear multiple regression analyses (one for each OQ dimension) to verify the hypothesis on the associations between psychological distress, social support, and dyadic adjustment.

The stepwise conditional method was employed, and the variables were inserted into the regression equation in two blocks. First, only age, relational status (single/couple), and self-definition of gender identity (AFAB, AMAB) were entered (model 1), whereas the dimensions of social support (i.e., support from family, friends, and a significant other) were added as predictors in the second step (model 2). No correlations more than 0.80 and no problems with multicollinearity (VIF < 2.5 for each independent variable) were observed for regression analyses.

##### OQ—Total

For the total sample, model 2 was found significant (*F* = 6.44, *p* < .001). The independent variables explained 41.4% (adjusted *R*^2^ = .414) of variance in the total score for psychological distress. Age (*β* = − .331, *p* < .001), family support (*β* = − .313, *p* < .005), and dyadic adjustment (*β* = − .376, *p* < .001) were significantly related to the total score for OQ-45.2 (Table 3). The results indicate that higher levels of psychological distress were associated with lower age and lower levels of family support and dyadic adjustment.

**Table 3:** Age, self-definition, relational status, social support, and dyadic adjustment regressed on psychological distress (*N* = 102)

Model 2	Variables	*B*	*SE*	*β*	*t*	*p*	*F*
	Age	−.733	.256	−.331*	−2.868	.006	1.225
	Self-definition	−8.917	6.180	−.170	−1.443	.156	1.285
	Relational status	−11.376	6.271	−.220	−1.814	.076	1.354
	Family support	−1.259	.525	−.313*	−2.397	.021	1.575
	Friends support	−.944	.647	−.171	−1.460	.151	1.265
	Significant other support	1.097	.873	.184	1.256	.215	1.988
	Dyadic adjustment	−.724	.192	−.482**	−3.761	.000	1.515

**p < *.001*; *p < *.05*

##### OQ—Symptom Distress

Model 2 was found significant for the total sample (*F* = 5.52, *p* < .001). The independent variables explained 36.6% (adjusted *R*^2^ = .366) of variance in the score for symptom distress. Age (*β* = − .366, *p* < .005), family support (*β* = − .272, *p* < .05), and dyadic adjustment (*β* = − .533, *p* < .001) were significantly related to symptom distress (Table 4). The result indicates that higher levels of symptom distress were associated with young age and lower levels of family support and dyadic adjustment.

**Table 4:** Age, self-definition, relational status, social support, and dyadic adjustment regressed on symptom distress (*N* = 102)

Model 2	Variables	*B*	*SE*	*β*	*t*	*p*	*F*
	Age	−.513	.166	−.366*	−3.083	.003	1.223
	Self-definition	−6.133	4.038	−.185	−1.519	.135	1.289
	Relational status	−3.430	4.088	−.105	−.839	.406	1.353
	Family support	−.695	.343	−.272*	−2.027	.048	1.563
	Friends support	−.621	.423	−.178	−1.467	.149	1.273
	Significant other support	.854	.571	.227	1.494	.142	1.997
	Dyadic adjustment	−.505	.124	−.533**	−4.059	.000	1.495

**p < *.001*; *p < *.05*

##### OQ—Interpersonal Relationships

The study found that model 2 was significant (*F* = 11.4, *p* < .001) for the total sample. The independent variables explained 57% (adjusted *R*^2^ = .570) of variance in the score for interpersonal relationships distress. Age (*β* = − .223, *p* < .05), self-definition (*β* = − .204, *p* < .05), relational status (*β* = − .235, *p* < .05), family support (*β* = − .223, *p* < .05), friend support (*β* = − .209, *p* < .05), and dyadic adjustment (*β* = − .514, *p* < .001) were significantly associated with interpersonal relationships distress (Table 5). This means that younger trans women who receive little support from family and friends and have low levels of dyadic adjustment are more likely to experience discomfort in interpersonal relationships.

**Table 5:** Age, self-definition, relational status, social support, and dyadic adjustment regressed on interpersonal relationships distress (*N* = 102)

Model 2	Variables	*B*	*SE*	*β*	*t*	*p*	*F*
	Age	−.135	.059	−.223*	−2.285	.027	1.223
	Self-definition	−2.940	1.439	−.205*	−2.043	.047	1.289
	Relational status	−3.428	1.457	−.242*	−2.353	.023	1.353
	Family support	−.273	.122	−.247*	−2.233	.030	1.563
	Friends support	−.316	.151	−.209*	−2.092	.042	1.273
	Significant other support	.110	.204	.067	.540	.592	1.997
	Dyadic adjustment	−.228	.044	−.556**	−5.145	.000	1.495

**p < *.001*; *p < *.05*

##### OQ—Social Role Functioning

For the total sample, model 2 was non-significant (*F* = 11.4, *p* > .05).

## Discussion

The study was conducted within a theoretical framework that considered the connection between the psychological well-being of transgender individuals and the quality of family/social support and couple relationships. The study draws from the premise that the links between psychological symptoms and transgenderism are not inevitable, and that the greater psychological vulnerability of the transgender condition can be understood only through a careful analysis of the relational contexts of the sense of belonging of transgender people.

Psychological symptoms are the outcome of discrimination experienced in family and social contexts or low levels of support and are not directly dependent on transgender identity.

In line with these considerations, the comparison between the mean scores in the OQ-45.2 obtained in the sample and the cutoff scores of the Italian adaptation of OQ-45.2 revealed that transgender individuals exhibited lower scores than the normative clinical sample. This finding is in line with recent findings that revealed no significant differences in the levels of depressive symptoms and physical and mental well-being between transgender and cisgender women (Vedovo et al., 2021).

However, the data also revealed higher scores with respect to the normative non-clinical sample in terms of symptoms, social relationships, and social roles. The study interprets this data as referring to the assumptions of minority stress theory, which posits that mental health disparities between marginalized individuals and their privileged counterparts are due to chronic exposure to stressors, such as stigma, prejudice, and lower levels of support (Hendricks & Testa, 2012; Meyer, 2003; Testa et al., 2015). Indeed, various studies revealed that transgender individuals report difficulties in interpersonal relationships (Nobili et al., 2018; Stewart et al., 2018) and challenges within family dynamics (Dierckx et al., 2017).

Consistent with the scientific literature analyzed, the current data confirm that the support and acceptance of one’s partner and family of origin play a crucial role in promoting well-being and represent an important protective factor in terms of negative health outcomes (Ryan et al., 2010; Seibel et al., 2018). Furthermore, the data revealed that higher levels of global psychological distress, symptom severity, and interpersonal relationship distress were associated with lower levels of family support and dyadic adjustment. The importance of couple relationships on the examined variables also emerged in the comparison between singles and individuals engaged in a couple relationship, where the singles displayed significantly higher levels of psychological distress.

In addition, higher levels of interpersonal relationship distress were found among those who reported lower levels of support from friends and who are women and younger transgender individuals. The findings related to interpersonal relationship distress confirmed those of previous research that found that younger transgender participants reported higher rates of prevalence of mental health diagnoses (Hyde et al., 2013) and psychological distress symptoms (Bockting et al., 2013; Bariola et al., 2015). Moreover, such participants discussed that trans women experienced more difficulties in interpersonal and romantic relationships (Iantaffi & Bockting, 2011; Lewins, 2002).

In summary, the study provides evidence that is consistent with the empirical literature (e.g., Bischof et al., 2011; Joslin-Roher & Wheeler, 2009; Simons et al., 2013a, b) and confirms that acceptance and support from one’s parents and family play a crucial role in promoting well-being among transgender individuals. The reason underlying this result may be that they are fundamental components in the processes of gender affirmation and acceptance of one’s gender identity throughout development (Seibel et al., 2018).

Through family ties, individuals build a sufficiently stable sense of self, which is expressed in the ability to feel a sense of continuity with one’s past and to differentiate oneself from it without feeling an implied loss of meaningful ties at the same time. In relationships with significant others, such as family, partner, and close friends, transgender individuals can build and witness the recognition of their identity (Grant et al., 2011; Soares et al., 2011). Therefore, the result that those who perceive lower levels of support perceived from partners and families experience increased difficulty in their existential condition and present higher levels of psychological distress is not surprising.

The current data revealed that the couple relationship represents an important protective factor for partners’ psychological well-being. Firstly, because people in couple relationships seem to present significantly lower levels of distress than single individuals and because the levels of perceived dyadic adjustment, which is understood as the synthesis of the levels of cohesion, consensus, satisfaction, and affective expression within the dyad, represent the most powerful predictor of levels of psychological distress.

The results are not surprising given that, in many cases, the partner represents the only source of support and comfort for transgender individuals, where their contexts tend to isolate, judge, and stigmatize (Marshall et al., 2020). The findings suggest that being in an intimate relationship with a partner may help transgender individuals to feel protected and worthy of love in the face of the relational difficulties associated with their condition (Meier et al., 2013).

### Limitations and Future Directions

Certain limitations of the study should be highlighted. The first includes the sample size and non-probability sampling. Therefore, caution is warranted regarding the interpretation of the findings. Moreover, further studies should be conducted on larger samples to improve the generalizability of the results. The data are cross-sectional and, therefore, the study cannot confirm causal associations. As such, future longitudinal studies are required to determine the cause–effect relationships between social support, dyadic adjustment, and psychological well-being.

A further limitation is that we relied solely on self-report data. It is thought that responses to questions about sensitive issues, such as the quality of intimate relationships, can be influenced by cognitive biases such as denial, idealization, and social desirability bias. This could be addressed through qualitative research and administration of semi-structured interviews for providing data that are more accurate.

Also, although the DAS has been used in some studies examining couple relationships in transgender individuals (Gamarel et al., 2014; Tornello, 2020), it was developed without consideration of a specific transgender population. Future studies could use methodologies and instruments that better analyzed the complexity and challenges that these new family realities bring.

Moreover, reflecting on the moderating or mediating role of variables, such as internalized stigma, positive and negative feelings toward transgender identity, and internalized transphobia on psychological distress is an interesting avenue for further research.

In addition, although recent studies revealed that trans women appeared to experience stronger social stigma and psychological distress than trans men (Stanton et al., 2021; Tan et al., 2020; Verbeek et al., 2020), the current study did not find significant differences in the levels of psychological distress between transgender men and women. This finding may be explained by the size of the sample, which warrants further investigation.

Finally, the sample size and the nature of the sampling did not allow for an adequate reflection on the differences between those who transitioned surgically or only socially; individuals who formed a family and had children before the transition and those who did not; individuals with different socio-cultural status and belonging to different ethnic groups; and binary and non-binary individuals. Information was obtained through an online survey in collaboration with associations active in the protection of the rights of transgender individuals. This scenario may have distorted the results as persons without access to the Internet or uninvolved in the associative sector may have been excluded. Therefore, future studies should consider this limitation when using different forms of recruitment and overcoming accessibility barriers.

### Practical Implications

Among LGBTQI + individuals, transgender people have to face specific forms of discrimination in different social settings, including the family and the workplace. Moreover, the legislative framework brings upon trans people additional disadvantage. The discrimination experienced by trans people encourages and justifies transnegative attitudes (Turner-Frey, 2014), and has a direct negative impact on their well-being (Drydakis, 2022). Therefore, programs to prevent and support transgender individuals’ health are required (Drydakis, 2022) and policymakers should explicitly address the needs of trans people in national health strategies. Policies granting equal rights and prohibiting discrimination encourage a supportive climate, thus reducing stigma and stress toward trans individuals, protecting their physical and mental health (Hatzenbuehler et al., 2013).

Our results emphasize the need to develop specific interventions for trans individuals and their families and suggest that building family- and partner-centered policies and programs would be important for trans individuals to avoid the emotional and psychological costs of discrimination (Katz-Wise Rosario & Tsappis, 2016; Sidiropoulou et al., 2019).

Concerns expressed by trans clients in the therapy settings often result from institutionalized forms of discrimination (e.g., difficulty to obtain proper identity documents), thus highlighting the need for psychotherapists to address the source of the distress and not just the symptom (Edwards et al., 2019). This would promote the possibility that new narratives could be constructed in the clinical setting and then spread in the social context with a significant cultural impact.

These premises have a number of practical clinical implications. At first, it is necessary to consider how clinical settings and protocols may reinforce cisnormativity and pathologization of different gender identity expressions (Blumer et al., 2013; Drydakis, 2022).

In addition, given the centrality of family relationships and perceived support to the psychological well-being of transgender people, therapists and counselors can involve parents, partners, and siblings in clinical and counseling processes. This would allow them to work directly on family dynamics and create a context that fosters communication, sharing of experiences, difficulties, emotions, and mutual support. In this scenario, family members can access the deeper meanings of trans family members’ experiences. In turn, transgender people can improve their understanding of their family members’ unique experiences and their difficulties in providing support (Edwards et al., 2019; Norwood, 2012). Counselors need to create a safe environment and accept the different positions family members may take on conflicting issues, with the understanding that intense conflicts, if managed constructively, can be important tools through which mutual understanding and acceptance as a trans couple and/or family can be achieved (Giammattei, 2015).

Specific clinical interventions should also involve couples in which one partner is trans that may seek counseling not only for issues related to gender affirmation and expression but also for managing the psychological impact of minority stress (Gamarel et al., 2014; Giammattei, 2015). In this regard, therapists should be aware that the relational dynamics of trans people may be different from those typically expected of cisgender clients (Singh & Dickey, 2017). In addition, therapists should support trans people’s experience of adjustment and intimacy in contexts of cisgenderism and discrimination (Bischof et al., 2016; Pulice-Farrow et al., 2019; Riggs, et al., 2015a, b).

Finally, with specific regard to therapeutic intervention, a final implication concerns the need to include gender identity issues in therapeutic training programs. In Italy, in fact, there is a need to implement training programs for health professionals on trans health needs, cultural competence, and discrimination awareness (Dionisi et al., 2020; Vitelli et al., 2017).

Students rarely address gender identity issues during their training, which makes them particularly unconfident when encountering the experience of trans people in their clinical practice. In addition, the limited training offered and the need to independently explore these issues only when required make clinical work with trans clients seem like a subspecialty that all therapists are not expected to have. This often leads to trans clients being sent to colleagues considered “experts” in trans issues (Edward et al., 2019) without problematizing the ethical issue of referral based on client demographics (McGeorge et al., 2016).

As suggested by Edward et al. (2019), the psychological profession should also build on widespread training related to issues of gender identity and the individual, family, and social experience of trans people in order to promote these clinical skills as essential and not as an exception or subspecialty.

## Conclusions

Transgender individuals experience specific problems related to social support at different points during the process of gender affirmation. Frequently, many of them have a long history of broken relationships behind them (Giammattei, 2015). Interventions that view social support as a central protective factor and that promote better relationships between transgender individuals and their families may prevent their vulnerabilities and improve their quality of life (Seibel et al., 2018; Valentine & Shipherd, 2018). This scenario seems particularly promising for individual, couple, and family interventions that are configured to be affirmative care settings that embrace a non-dichotomous view of gender or gender norms (Malpas & Glaser. 2017). In this context, interventions should be respectful of all aspects of clients’ identities, beginning with their names and ending with experiences of discrimination, violence, and abuse, which many in the community may have directly experienced or witnessed (Giammattei, 2015).

The field of psychology continues to lack validated studies and practices for this population (Benson & Piercy, 2009; Seibel et al., 2018). In addition, clinicians have exhibited certain difficulties in planning interventions that are dedicated to transgender individuals and their significant others, which is in part explained by the infrequent inclusion of specific expertise on these issues in undergraduate and postgraduate specialty training programs (Biaggio et al., 2003).

Therefore, promoting and planning specific training courses would be desirable to prepare counselors and therapists for non-discriminatory health services that target transgender clients and their families to guarantee that they can exercise their right to comprehensive care as required by legislation.

## Data Availability

Not applicable.
